# Critical considerations in the administration of antineoplastic therapy for patients with malignant solid tumors undergoing hemodialysis

**DOI:** 10.3389/fonc.2026.1777042

**Published:** 2026-04-22

**Authors:** Jun Li, Qi Ke, Wen-qing Lv, Dao-yuan Lv, Jing-yao Lu

**Affiliations:** 1 Department of Nephrology, Affiliated Hospital of Jiangnan University, Wuxi, Jiangsu, China; 2 Wuxi School of Medicine, Jiangnan University, Wuxi, Jiangsu, China

**Keywords:** hemodialysis, malignant solid tumors, antineoplastic therapy, adverse effect, pharmacokinetics

## Abstract

Patients with chronic kidney disease exhibit a higher risk of developing malignancies, and among those with end-stage renal disease (ESRD) on dialysis, approximately 7% are diagnosed with new-onset tumors within five years. Patients with ESRD who are receiving antineoplastic treatment exhibit a significantly higher incidence and greater severity of adverse effects associated with antineoplastic agents compared to individuals with normal renal function. This complexity involves multiple factors, including the therapy options and dose adjustment of antineoplastic agents, drug-related toxicities, management of fluid overload, and the maintenance of internal homeostasis in ESRD patients with malignancies. Patients undergoing hemodialysis are at heightened risk of drug toxicity if doses of renally cleared medications are not appropriately individualized; conversely, subtherapeutic drug exposure may occur due to premature and substantial removal of medications during the dialysis session. Unfortunately, there is little information on the appropriate dosing and pharmacokinetics of most chemotherapeutic agents in dialysis patients being treated for cancer. Therefore, ensuring the safety of antineoplastic therapy in ESRD patients has emerged as a significant clinical challenge for both nephrologists and oncologists. This article summarized the effects of uremia and hemodialysis on the pharmacokinetics of commonly used antineoplastic agents, along with an overview of real-world evidence on the use of these agents in patients with ESRD undergoing hemodialysis. It aims to provide clinically valuable information to optimize antineoplastic therapy in this patient population, with the ultimate goal of prolonging survival periods and reducing the incidence of severe drug-related complications.

## 1 Introduction

Patients with chronic kidney disease exhibit a higher risk of developing malignancies (1), and among those with end-stage renal disease (ESRD) on dialysis, approximately 7% are diagnosed with new-onset tumors within five years (2, 3). However, patients with chronic kidney disease, particularly those with ESRD, are often excluded from clinical trials of antineoplastic agents. And this exclusion hinders the development of evidence-based treatment strategies and optimal, individualized chemotherapy dosing in this patient population (4).

Antineoplastic therapy in cancer patients with ESRD presents significant challenges. On one hand, these patients are at increased risk of developing extra-renal toxicity due to elevated systemic drug exposure; on the other hand, they may experience subtherapeutic drug concentrations as a result of premature and extensive drug elimination during dialysis. Unfortunately, there is little information on the appropriate dosing and pharmacokinetics of most chemotherapeutic agents in dialysis patients being treated for cancer. It is essential to propose an optimal anticancer drug strategy (dosage, timing, monitoring) to dialysis patients (whenever possible) rather than contraindicating it systematically. Therefore, dedicated pharmacokinetic studies in patients undergoing hemodialysis are warranted—particularly in those with cardiovascular or cerebrovascular comorbidities and in older adults. It is crucial to use the available data to adjust the dose of antineoplastic agents for these patients and to schedule the administration according to dialysis sessions. This article provides a comprehensive review of the impact of uremia and hemodialysis on the pharmacokinetics and metabolism of commonly used antineoplastic agents, supplemented by a synthesis of real-world evidence regarding their use in patients with end-stage renal disease undergoing hemodialysis. It aims to provide clinically valuable information to optimize antineoplastic therapy in this patient population, with the ultimate goal of prolonging survival periods and reducing the incidence of severe drug-related complications.

## 2 Epidemiology of patients with cancer undergoing hemodialysis

### 2.1 Higher incidence of malignancy in patients with uremia

It is estimated that by 2030, approximately 5.4 million people worldwide suffering from ESRD were undergoing some form of kidney replacement therapy, as reported by the CNRDS (5). Hemodialysis has been the predominant treatment modality for ESRD since 2012, accounting for 82.4% of incident ESRD cases in 2022 (6). The 5-year cumulative incidence of any cancer among 482,510 incident hemodialysis patients was 9.48%, with higher rates observed in specific subgroups, including older age, male, non-White individuals, non-Hispanic populations, those with a non-diabetic cause of end-stage renal disease (ESRD), recent initiation of hemodialysis therapy, a history of transplantation evaluation, those with acquired renal cystic disease, and an erythropoietin dose of 20,000 U/week or higher (7, 8). A retrospective analysis of data from 1,377 incident hemodialysis patients across seven hemodialysis centers in Eastern Europe revealed that 63% had a history of cancer prior to hemodialysis initiation, predominantly hematologic and gastrointestinal malignancies; in contrast, 6.89% were diagnosed with *de novo* cancers after the initiation of hemodialysis, primarily involving gastrointestinal cancers, kidney cancer, urothelial cancers, lung cancer, and thyroid cancer (2). In recipients of renal transplants, the incidence of malignancies linked to infections and immune dysfunction—including Kaposi sarcoma, non-Hodgkin lymphoma, Hodgkin lymphoma, lip cancer, and non-epithelial skin cancers—was higher during periods of functional graft compared to periods of graft failure (9, 10). The mean times for cancer development after the beginning of the dialysis sessions range from 1 to 5 years in different clinical research, with an even greater frequency observed within the first year (2, 7, 11–13). Another study investigating the risk of cancer recurrence among 4,912 dialysis patients with a history of neoplasia found that 323 (7%) experienced tumor recurrence, with a mean time to recurrence of 1.2 years from the initiation of dialysis; and 80% of these cases involved metastasis (3).

The carcinogenic factors associated with uremia and hemodialysis therapy primarily include impaired immune function (7, 14), oxidative stress and reduced antioxidant capacity (15), inadequate dialysis efficacy, accumulation of carcinogenic substances resulting from uremic toxins and the biological incompatibility of dialysis membranes, leaching of plasticizers from tubing materials (16), chronic microinflammation (17), and exposure to medications with potential carcinogenic risks (18–20). Furthermore, acquired renal cystic disease significantly increases the risk of developing renal cell carcinoma (18). The carcinogenesis factors triggered by uremia and hemodialysis treatment were listed in **Table 1** (7, 14–20).

**Table 1:** Carcinogenesis factors triggered by uremia and hemodialysis treatment.

Risk factors	Mechanism
Impaired immune function (7, 14)	Increased susceptibility to oncogenic viral infections, as evidenced by a higher incidence of Human Papillomavirus (HPV), Human Herpesvirus 8 (HHV-8), and Epstein-Barr Virus (EBV)-related malignancies
Oxidative stress and diminished antioxidant capacity (15)	Uremia, chronic microinflammation and the biological incompatibility of dialysis membranes and dialysis tubing (16)
Accumulation of carcinogenic agents (15)	The biological incompatibility of dialysis membranes, along with plasticizers released from tubing materials
Infection and chronic microinflammation (17)	Chronic microinflammation due to uremia and hemodialysis, Chronic hepatitis B, chronic hepatitis C
Insufficient dialysis efficacy	Accumulation of uremic toxins
Renal lesions predisposing to malignant tumor development (18)	Acquired renal cystic disease (18),Chronic aristolochic acid nephropathy (19)
Pharmacological factors (20)	Immunosuppressive agents, Erythropoietin, Analgesic abuse

### 2.2 Survival rate in dialysis patients with malignancy

Cancer was an independent predictor of elevated mortality risk among hemodialysis patients, with those diagnosed facing a sixfold greater risk compared to their cancer-free counterparts (2). In a large registry-based study from Australia and New Zealand, the standardized mortality rate (SMR), which is a measure of excess deaths in dialysis patients relative to the general population, was 2.6 for all cancers and 1.3 for *de novo* cancer, particularly kidney, colorectal, and lung cancers (21). Patients with recurrent cancers exhibited a 3-year survival rate of less than 50%, and the most common recurrent cancers included lymphoma, tumors of the urinary tract, lung cancer, and melanoma (22). A retrospective cohort study of 639 patients initiating hemodialysis between July 1999 and December 2017 demonstrated that the 1-, 5-, and 10-year cumulative survival rates following cancer diagnosis were 58.73%, 34.64%, and 20.41%, respectively (8). A retrospective study of 675 hemodialysis patients diagnosed with cancer revealed that effective chemotherapy could offer the potential for prolonged survival, even in cases of unresectable tumors (23).

Therefore, early screening and evidence-based optimization of antineoplastic treatment regimens are critical to improving both overall survival and quality of life in hemodialysis patients with concomitant malignancies.

## 3 The impact of malignant tumors and antineoplastic therapy on patients undergoing hemodialysis

### 3.1 Electrolyte and acid-base imbalance

Patients with cancer are especially susceptible to severe electrolyte disturbances resulting from paraneoplastic syndromes, surgical removal of endocrine organs, inadequate nutritional intake, tumor metastases, and concomitant chemotherapy agents such as platinum and ifosfamide (24), immune checkpoint inhibitors (ICIs), epidermal growth factor receptor inhibitors, and fibroblast growth factor inhibitors (25). Hyponatremia (26), hypocalcemia (27), hypokalemia, hypomagnesemia, hypercalcemia (28), and, less commonly, lactic acidosis (29) are frequently observed electrolyte disturbances that may occur in cancer patients. Notably, in ESRD patients undergoing hemodialysis, the common electrolyte abnormalities include fluid overload, hyperkalemia, hyperphosphatemia, and metabolic acidosis. Additionally, tumor lysis syndrome is unique to cancer patients and can be associated with acute kidney injury, acute uric acid nephropathy and multiple electrolytes disturbances, including hyperkalemia, hyperphosphatemia, hypocalcemia and hypermagnesemia.

Therefore, electrolyte disturbances and metabolic acidosis should be closely monitored in hemodialysis patients with malignancy, particularly those receiving antineoplastic agents that may aggravate such disturbances.

### 3.2 Malnutrition and infection

Patients with advanced malignancies often suffer from malnutrition and infections, which contribute to reduced tolerance to chemotherapy, a condition further exacerbated by ESRD. Moreover, hypoproteinemia affects the binding and metabolism of chemotherapy drugs. Besides, malnutrition, the administration of chemotherapy drugs, and gastrointestinal bleeding all exacerbate the condition of anemia in cancer patients undergoing hemodialysis.

### 3.3 High incidence of cardiovascular events

Cancer-associated hypertension—including paraneoplastic syndromes driven by excessive vasoactive peptide secretion (30), certain antineoplastic agents (31, 32), and nonsteroidal anti-inflammatory drugs—is associated with an elevated risk of major adverse cardiovascular events, including myocardial ischemia, heart failure, stroke, and cardiovascular death. Cancer-associated thrombotic microangiopathy—driven by mucin-producing malignancies (33) and select chemotherapeutic agents (34)—mediates microvascular endothelial injury, thereby triggering platelet activation, fostering microthrombus formation, amplifying blood pressure lability, and worsening anemia; these interrelated pathophysiological processes collectively accelerate cardiovascular event development. Furthermore, Certain antineoplastic agents exhibit cardiotoxic properties. These factors contribute to an increased incidence of cardiovascular events in patients with ESRD.

**Figure 1:**
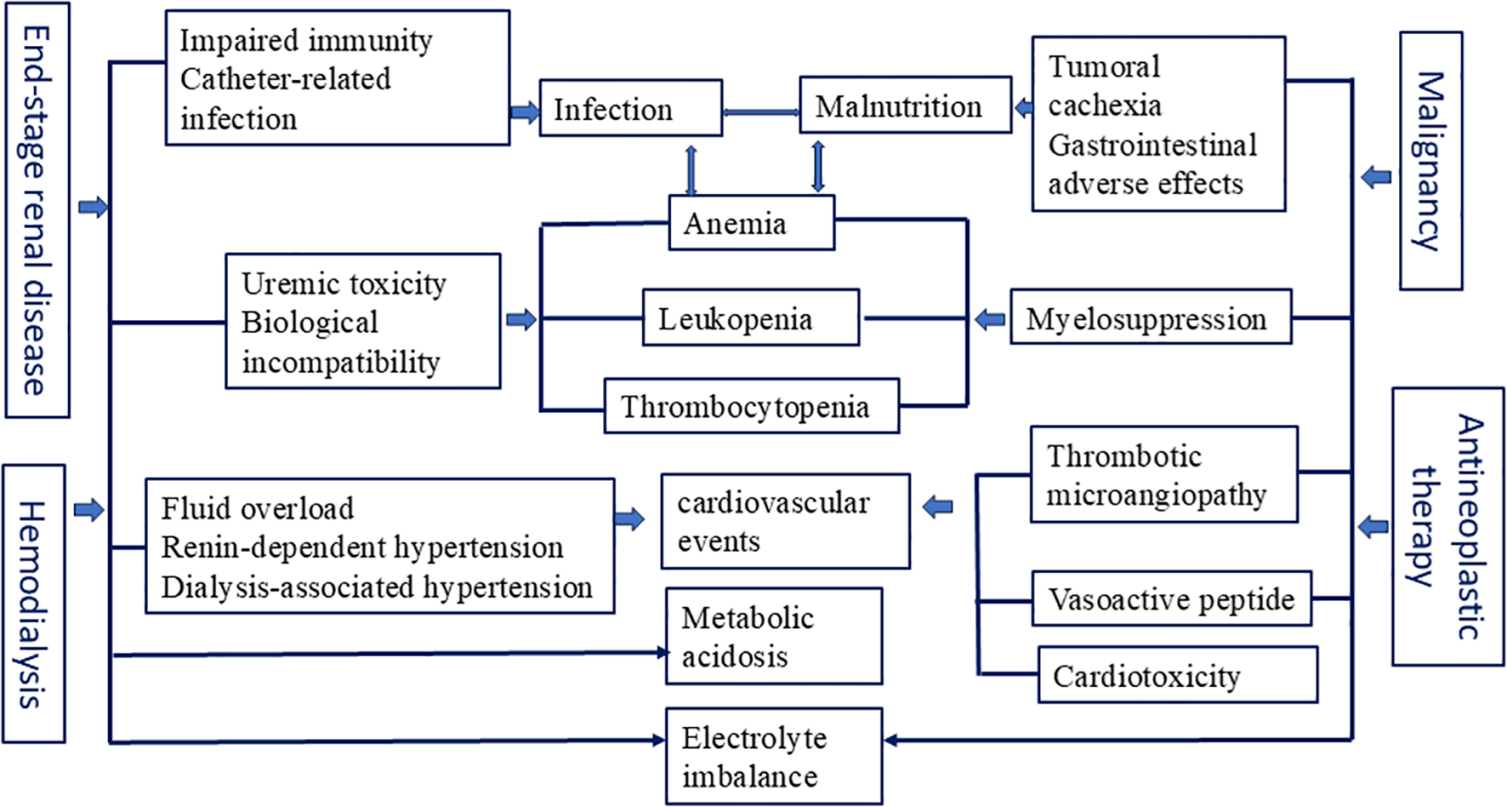
The impact of malignant tumors and antineoplastic therapy on patients undergoing hemodialysis.

Moreover, opioid prescribing for patients with malignancy undergoing hemodialysis must account for uremic physiology as well as the degree of renal excretion and drug clearance achieved during hemodialysis sessions (35). The potential cardiovascular adverse effects and the risk of febrile neutropenia should be closely monitored in older patients with ESRD receiving antineoplastic therapy (36). The impact of malignant tumors and anti-tumor treatments on hemodialysis patients is shown in the **Figure 1**. 

## 4 The influence of uremic and hemodialysis on drug metabolism

### 4.1 The increased volume of distribution of drugs in hemodialysis patients

Chemotherapeutic drugs that exhibit a high volume of distribution are generally less accessible to dialysis compared to those with a low volume of distribution, as the latter are more likely to be restricted to the intravascular space (37). This abnormality may be due to fluid overload, decreased protein binding, or altered tissue binding.

### 4.2 The influence of drug metabolites and molecular weight on dialysis clearance

Drugs with a molecular weight less than 1kDa can diffuse through membranes. However, drugs that primarily bind to plasma proteins are poorly amenable to dialysis due to the high molecular weight and structural characteristics of the drug-protein complex (37). Acidic medications generally exhibit binding affinity toward albumin. Consequently, in patients with severe renal impairment, reduced plasma protein levels may increase the concentration of unbound drug, thereby enhancing its elimination. Basic drugs exhibit a high affinity for alpha1-acid glycoprotein. Given the elevated levels of this acute-phase reactant in hemodialysis patients—where it is predominantly bound to basic substances—the administration of cationic drugs may result in increased free drug concentrations, leading to enhanced pharmacological effects and an increased risk of dose-dependent adverse events (38). Moreover, elimination through dialysis is also influenced by the transfer rate of the drug from tissues to plasma; specifically, a shorter plasma half-life facilitates a higher transfer rate, thereby enhancing the dialyzability of the drug. FHD has been recommended as an indicator associated with drug dialyzability; when FHD exceeds 25%, the drug should be administered after dialysis (39).

Notably, another factor concerns the transformation of drugs into active or inactive metabolites that may have different pharmacokinetic characteristics compared with the parent drug.

### 4.3 Abnormal liver metabolism

Chemotherapeutic drugs mainly metabolized in the liver via CYP3A4, CYP2D6, or cytochrome P450 enzymes in the liver. For patients with concomitant liver injury or those with underlying hepatic conditions such as liver cancer and cirrhosis—both of which are associated with reduced hepatic reserve—special attention should be paid to the impact of impaired liver function on drug metabolism.

Hence, a customized strategy for each medication, informed by their pharmacokinetic profiles in hemodialysis patients, is crucial for optimizing patient outcomes while minimizing potential risks in this susceptible population.

## 5 The administration of antineoplastic agent in hemodialysis patients

Antitumor drugs are primarily categorized into three main classes: traditional cytotoxic chemotherapeutic agents, targeted chemotherapeutic agents, and immunotherapies for tumor treatment. The summary of antineoplastic agents in hemodialysis patients is presented in **Table 2**.

**Table 2:** The summary of antineoplastic agents in hemodialysis patients.

Classification of antineoplastic agents	Antineoplastic agents	Renal excretion	Hemodialysis clearance	Dose adjudgment in ESRD patients	Research type	Adverse effect
Traditional cytotoxic chemotherapeutic agents	Cisplatin	Mainly renal excretion	Yes	25-50% of the standard dose 25-50mg/m^2^	multicenter retrospective study (23, 43–45)	Myelosuppression, Neurotoxicity, ototoxicity, hypersensitivity reactions, Gastrointestinal reaction, hypomagnesemia, hyponatremia, and Fanconi syndrome
	Carboplatin	Mainly renal excretion	Yes	AUC × 25 100-150mg	multicenter retrospective study (23, 41, 42)	Myelosuppression, hepatotoxicity, Gastrointestinal reaction, hypersensitivity reactions,
	Oxaliplatin	Mainly renal excretion	Yes	25%–50% of the standard dose 65mg/m^2^	Caseseries (23, 46, 58).	Neurotoxicity, gastrointestinal reaction, hypersensitivity reactions, hepatotoxicity, myelosuppression
	Irinotecan	<20%	irinotecan was partially dialyzable, SN-38 was not dialyzable	weekly dose of 50 mg/m²	Case reports (23, 50)	Neutropenia, Severe Diarrhea, hepatotoxicity
	Etoposide	40%	No	50–60% dose reduction, 50mg/m^2^, escalate incrementally to 100 mg/m^2^.	Single-center pharmacokinetic study (43, 52, 53)	hematological toxicity, gastrointestinal reaction
	5-fluorouracil (5-FU)	15%	Yes (90%)	standard dose or appropriate dose reduction (10-20%) 300-500mg/m^2^, intravenous drip	Multicenter retrospective study (23, 54, 55)	lactic acidosis, hyperammonemia, myelosuppression, neurotoxicity, Hand-foot syndrome, Mucositis and gastrointestinal toxicity, cardiotoxicity
	Capecitabine	Mainly renal excretion	Not available	50%-75% dose reduction 600mg/m^2^, twice daily	Case reports (58, 60)	Myelosuppression, neurotoxicity, gastrointestinal toxicity, hand-foot syndrome, cardiotoxicity
	Gemcitabine	<10%	Partly eliminate dFdU	standard dose or appropriate dose reduction 1000 mg/m^2^	Multicenter retrospective study (23, 62–64)	HUS, Myelosuppression, pulmonary toxicity, hypersensitivity reactions, cardiac arrhythmias.
	Vinorelbine	8%	Not available	30% to 50% dose reduction 25mg/m^2^	Case reports (67)	Myelosuppression, phlebitis, gastrointestinal reactions, cardiotoxicity
	Paclitaxel	<10%	No	75–150 mg/m²	Multicenter retrospective study (23, 40, 43, 69)	peripheral neuropathy, neutropenia, hypersensitivity reactions, cardiovascular Events
	Docetaxel	insignificant	No	65 mg/m²	Multicenter retrospective study (23, 43, 44, 70)	fluid overload, the remaining adverse effects are consistent with those of paclitaxel
	Actinomycin D	30%	No	20% to 30% dose reduction 02-0.3 mg/m²	Case reports (72)	Myelosuppression, hepatotoxicity, gastrointestinal reactions
	Doxorubicin	15%	No	65 mg/m^2^	Multicenter retrospective study (23, 43, 75)	Myelosuppression, cardiotoxicity, gastrointestinal reactions
	Epirubicin	10%	No	30mg/m^2^	Case reports (44, 73)	Myelosuppression, cardiotoxicity, gastrointestinal reactions
	Cyclophosphamide (CTX)	50-70%	Yes (22%)	25-30% dose reduction 0.5-0.8g/m²	Case series (44, 78)	Myelosuppression, hepatotoxicity, reproductive toxicity, bladder toxicity, gastrointestinal reactions
	Ifosfamide	70-80%	Yes (87%)	1.5–2 g/m²	Case series (78, 79)	Myelosuppression, bladder toxicity, neurotoxicity, gastrointestinal reactions
Targeted chemotherapeutic agents	PARP inhibitors- Olaparib	15%	No	Dose reduction 200 mg bid	Case reports (82)	hematologic toxicity, gastrointestinal reactions
	Bevacizumab	insignificant	No	50% dose reduction 5 mg/kg	Case series (84, 85)	Hypertension, TMA, gastrointestinal tract perforation, hemorrhage, cardiotoxicity
	sunitinib	<20%	No	dose reduction 25–50 mg/d	Multicenter retrospective study (23, 84–88)	Hypertension, hand–foot syndrome, mucosal inflammation, hypothyroidism gastrointestinal reactions, cardiotoxicity, hepatotoxicity
	Axitinib	<25%	No	6-12mg/d	Case series (23, 84)	Hypertension, hand–foot syndrome, mucosal inflammation, gastrointestinal reactions, cardiotoxicity, hepatotoxicity
	Sorafenib	<20%	No	100-800mg/d	Multicenter retrospective study (23, 84, 85)	Consistent with those of Axitinib
	Pazopanib	<20%	No	200-400mg/d	Case series (84, 85)	Consistent with those of Axitinib
	EGFR-TKIs	No	No	standard dose or appropriate dose reduction	Multicenter retrospective study (23, 43, 95, 96)	hematologic toxicity, gastrointestinal reactions, febrile neutropenia, cutaneous toxicity, cardiotoxicity
	BCR-ABL inhibitors- Imatinib	13%	No	400 mg/day	Case reports (23, 97, 98)	Edema, muscle cramps, nausea, diarrhea, and cutaneous reactions
	mTOR inhibitors	insignificant	No	standard dose	Retrospective study, single-center study (23, 24, 43, 100, 101)	Stomatitis, nausea, diarrhea, and cutaneous reactions, infection
	Anti-HER2-monoclonal antibodies	insignificant	No	standard dose	Case reports (23, 103, 104)	cardiotoxicity, hematological toxicity, and infusion-related reactions, cutaneous reactions
	BRAF inhibitors Vemurafenib	insignificant	No	standard dose	Case reports (107)	Hyperkeratosis, headache, fever, arthralgia, papilloma
	dabrafenib	insignificant	No	dose reduction 75-150mg/d	Case reports (105, 106)	Cytokine release syndrome, the remaining adverse effects are consistent with those of vemurafenib
	CDK 4/6 inhibitor Palbociclib	insignificant	No	dose reduction 100mg/d	Case reports (109)	Myelosuppression, cardiotoxicity, hepatotoxicity, Interstitial lung disease, venous thromboembolism, diarrhea
	Selective estrogen receptor modulator tamoxifen	insignificant	tamoxifen 25% N-desmethyl tamoxifen 27%	standard dose	Case reports (23, 110, 111)	endometrial lesions, cardiovascular events, and osteoporosis, hepatotoxicity, venous thromboembolism
	anastrozole		19%	standard dose	Case report (111)	
Immunotherapies for malignancies	Immune checkpoint inhibitors	insignificant	No	standard dose	Case series (84, 116, 119)	Immune-related adverse events
	CAR-T therapy	insignificant	No	standard dose of CAR-T dose Dose reduction of lymphocyte-depleting chemotherapeutic agents	Case reports (125, 126)	Cytokine release syndrome, immune effector-associated neurotoxicity syndrome and tumor lysis syndrome

AUC, Area Under Curve; HUS, Hemolytic uremic syndrome; TMA, thrombotic microangiopathy; ESRD, End-stage renal disease; PARP, inhibitors Poly (ADP-ribose) polymerase inhibitors; EGFR-TKIs, Epidermal growth factor receptor tyrosine kinases inhibitor; BCR-ABL, inhibitor Breakpoint Cluster Region-Abelson Leukemia Virus Inhibitors; mTOR, Inhibitors Mammalian inhibitor Target of Rapamycin inhibitors; HER2-monoclonal antibodies, Human Epidermal Growth Factor Receptor (HER2)-monoclonal antibodies; BRAF, inhibitors B-Raf proto-oncogene, serine/threonine kinase inhibitors; CDK 4/6 inhibitor, Cyclin-dependent kinase 4/6 inhibitor; CAR-T, therapy Chimeric antigen receptor T cell therapy.

### 5.1 Traditional cytotoxic chemotherapeutic agents

#### 5.1.1 Platinum antineoplastic agents

Carboplatin, cisplatin and oxaliplatin are mainly renal excretion and dialyzable through hemodialysis. Hemodialysis can eliminate platinum antineoplastic agents contributing to its utility as a treatment modality for platinum drug overdose.

Carboplatin is recommended to be based only on pharmacokinetics yet not calculated in relation to body surface. In patients undergoing hemodialysis, the dose that can be safely administered is the area under the concentration-time curve (AUC) 3.5–5 mg/ml·min (40). Another case report suggests that higher doses of carboplatin, administered with a shorter interval between the end of infusion and the initiation of hemodialysis (1 to 2 hours), may also be feasible in the therapy of ovarian cancer (23, 41, 42). Cisplatin-based chemotherapy can be administered to patients with advanced ovarian germ cell tumors and renal insufficiency at reduced doses (50–75% of the standard dose) to minimize adverse effects while maintaining efficacy within a multidisciplinary treatment framework (23, 43–45). Oxaliplatin is recommended to be administered at a reduced dose of 25%–50% after dialysis, with the dosing interval extended to three weeks (23, 46). Notably, hypomagnesemia, hyponatremia, and Fanconi syndrome should be closely monitored in hemodialysis patients with residual renal function who are receiving platinum-based antineoplastic therapy (47). In addition, targeted delivery of platinum-based antineoplastic agents may reduce the incidence of adverse effects. TPNPs are a promising antitumor agent as a self-assembled nanoprodrug with high drug loading capacity. As a cisplatin and tolfenamic acid-based prodrug, these self-assembled nanoparticles have been shown to improve therapeutic efficacy in a human ovarian cancer xenograft mouse model while reducing systemic toxicity (48).

#### 5.1.2 Irinotecan

Irinotecan is converted to an active metabolite, SN-38 and urinary excretion accounts for <20% of the elimination of the administered dose. Pharmacokinetic evaluation demonstrated that patients with severe chronic kidney disease exhibited higher unbound levels of SN-38 relative to individuals with normal renal function, primarily due to the suppression of SN-38 protein binding by the uremic toxin 3-carboxy-4-methyl-5-propyl-2-furanpropanoate (49). A noncompartmental pharmacokinetic analysis indicates that irinotecan exhibits partial dialyzability, whereas SN-38 appears to be nondialyzable (50).Thus, a lower weekly dose of 50 mg/m² of irinotecan may be more suitable to minimize the risk of severe neutropenia and infections, ideally administered following hemodialysis or on days without dialysis (23, 50). UGT1A polymorphisms, including variant alleles of UGT1A1*28, UGT1A1*60 and UGT1A9*22, may lead to accumulation of SN-38 in patients with ESRD, which may result in the lethally neutropenia (51).

#### 5.1.3 Etoposide

Approximately 40% of etoposide dose is excreted by the kidneys. Etoposide is not removed by hemodialysis and it can be used before or after hemodialysis sessions (43). Research has shown increased AUC and extended half-life of etoposide in patients, leading to recommendations for a 50%–60% dose reduction prior to hemodialysis to mitigate the risk of hematological toxicity (52). A dose-escalation trial involving five patients received etoposide at an initial dose of 50 mg/m² in combination with cisplatin prior to hemodialysis, demonstrating manageable adverse effects (53).

#### 5.1.4 5-FU and 5-FU-related chemotherapeutic drug

##### 5.1.4.1 5-fluorouracil

5-fluorouracil (5-FU) and its metabolite, fluorodeoxyuridine monophosphate, inhibits explicitly thymidylate synthase, which is crucial for synthesizing the thymidine necessary for DNA replication. 5-FU, a molecular entity weighing 130 Da, is mainly catabolized in the liver and a mere 15% *via* the urine in its original form. During hemodialysis session, around 90% of 5-FU and its metabolites-α-fluoro-β-alanine (FBAL) were directly cleared from the blood (54). 5-FU has been successfully used in hemodialysis patients with advanced hepatocellular carcinoma, esophageal cancer, and colon carcinoma (23, 55). Nevertheless, cases of lactic acidosis and hyperammonemia associated with 5-fluorouracil have been observed in cancer patients, and hemodialysis has proven to be an effective therapeutic intervention (56, 57). Myelosuppression is frequently observed, which can lead to reduced blood cell counts and increased risks of infection, anemia, and bleeding.

##### 5.1.4.2 Capecitabine

Capecitabine, with a molecular weight of 359.3 Da, is a prodrug of 5-fluorouracil (5-FU), which is metabolized in the liver to 5’-deoxy-5-fluorocytidine and subsequently to 5’-deoxy-5-fluorouridine (5’-DFUR), then converted to the active metabolite 5-FU within tumor tissues. This prodrug is processed and excreted mainly through renal pathways, with 96% of its dosage identifiable in the urine. Pharmacokinetics study demonstrated that administration of capecitabine is contraindicated in patients with a GFR below 30 mL/min, as a phase II study demonstrated a higher frequency of grade 3 and 4 adverse events in this population (58, 59). However, case reports of capecitabine support a recommended dose reduction of 50% to 75% in patients undergoing hemodialysis, which has demonstrated both good tolerability and efficacy in individuals with malignancies on hemodialysis (58, 60). Myelosuppression and cardiotoxicity necessitate careful dose management and vigilant medical supervision.

##### 5.1.4.3 Gemcitabine (2’,2’-difluorodeoxycytidine)

The primary metabolite of gemcitabine, difluorodeoxyuridine (dFdU), which is non-cytotoxic, exhibits an extended half-life and remains detectable in plasma for multiple days, even following minimal doses of the drug (61). The renal elimination of gemcitabine and its metabolites contribute <10% and 90%, respectively, to their complete removal. Pharmacokinetic parameters of gemcitabine were not altered, but a five-to tenfold prolongation of terminal half-life as well as a higher AUC of dFdU were found in a patient on hemodialysis (62). Given that hemodialysis is effective in lowering plasma levels of dFdU, it is recommended to start the procedure within 6 to 12 hours following gemcitabine administration (23, 63). A case report demonstrated the efficacy and safety of combination chemotherapy with full-dose gemcitabine (1,000 mg/m²) and reduced-dose cisplatin (35 mg/m²) in a patient with ESRD who had metastatic renal allograft collecting duct carcinoma (CDC) (64). Due to the heightened risk of severe adverse drug reactions (ADRs) in individuals with dihydropyrimidine dehydrogenase deficiency (DPYD)—a genetic variant that impairs fluoropyrimidine metabolism—screening for dihydropyrimidine dehydrogenase (DPD) deficiency prior to treatment is advised before starting fluoropyrimidine-based chemotherapy (65). Phenotypic assessment using the plasma [UH2]:[U] ratio is advised for patients with ESRD (66).

#### 5.1.5 Vinorelbine

Vinorelbine is mainly eliminated through the liver, with only 8% of the administered dose recovered unchanged from the urine. A 30% to 50% dose reduction of vinorelbine has been shown to be effective and well-tolerated in hemodialysis patients with Hodgkin lymphoma or metastatic breast cancer, helping to prevent adverse effects such as myelosuppression (67).

#### 5.1.6 Paclitaxel and docetaxel

The renal removal of paclitaxel and docetaxel is insignificant. Both drugs strongly bind to proteins such as albumin and alpha-1-glycoprotein, and not dialysable. Case reports indicate that paclitaxel doses ranging from 75 to 150 mg/m²—and in some instances exceeding this range—were administered safely to 16 patients with ovarian cancer undergoing maintenance hemodialysis, without necessitating dose modification attributable to renal impairment (23, 40, 43, 68, 69). A reduced dose of 65 mg/m² of docetaxel is recommended for cancer patients undergoing hemodialysis (23, 43, 44, 70). The common side-effect of paclitaxel is peripheral neuropathy, neutropenia, and hypersensitivity reactions. Notably, paclitaxel-coated balloons (AcoArt Orchid) have demonstrated both efficacy and safety in the treatment of dysfunctional arteriovenous fistulas in hemodialysis patients (71).

#### 5.1.7 Actinomycin D

About 30% of actinomycin D is excreted via urine and a standard dose can be given with precaution in patients with ESRD. It is not dialysable; hence, it can be given before or after dialysis. The primary concern associated with administering actinomycin D to patients undergoing hemodialysis is its propensity to induce myelosuppression; therefore, a 20–30% dose reduction is recommended—based on evidence from case report in end-stage renal disease (72).

#### 5.1.8 Doxorubicin and epirubicin

Doxorubicin and epirubicin are removed mainly by the liver and, to a lesser degree, are excreted by the kidneys (15% and 10%, respectively). Case reports documented patients with early-stage breast cancer undergoing hemodialysis who received epirubicin well, without severe adverse reactions (44, 73). However, pharmacokinetic studies showed that the AUC of doxorubicin and its active metabolite doxorubicinol was approximately 1.5-fold and 3-fold higher, respectively, in patients undergoing hemodialysis compared to those with normal renal function, with a similar elevation in AUC also observed for epirubicin (74). Doxorubicin administered at a reduced dose of 65 mg/m² was well tolerated in patients undergoing hemodialysis, despite concurrent leukopenia and thrombocytopenia (23, 43, 75).Therefore, acute myelosuppression and cumulative dose-related cardiotoxicity should be routinely monitored in hemodialysis patients receiving doxorubicin or epirubicin, as such monitoring forms the basis for dose adjustment. Doxorubicin bound to albumin through self-assembling dyes acting as Specific Polymolecular Ligands represents a promising immunotargeted therapy that may reduce adverse effects while enhancing doxorubicin accumulation in tumor tissue (76).

#### 5.1.9 Cyclophosphamide and ifosfamide

Cyclophosphamide (CTX) is inactive until processed by the liver into a number of active metabolites, which are eliminated through the kidneys. Approximately 50% to 70% of CTX is excreted by kidneys within 48 hours, with approximately 32% expelled in its unchanged form. The AUC was increased in hemodialysis patients and it is necessary to reduce the dose of CTX by 25-30% in hemodialysis patients. Approximately 22% of the drug was removed during the first three hours of dialysis, with an average total clearance rate lower compared to individuals with normal kidney function (70, 77). Therefore, a 25–30% dose reduction of CTX is recommended in patients undergoing hemodialysis (44, 78). Ifosfamide is mainly processed in the liver. The prodrug is converted into the active metabolite phosphoramide mustard, but it also produces the urotoxic compound acrolein as well as the neurotoxic and nephrotoxic metabolite chloracetaldehyde. *In vitro* studies suggest that hemodialysis can decrease ifosfamide concentrations by 87% and chloracetaldehyde by 77%. Three hemodialysis patients received individually adjusted doses of ifosfamide (initial dose: 1.5 -2g/m²), with myelosuppression identified as the most frequent adverse event and no instances of life-threatening toxicity observed (78, 79). Notably, both CTX and ifosfamide are associated with increased bladder toxicity, thereby elevating the risk of urinary system malignancies (80).

### 5.2 Targeted chemotherapeutic agents

#### 5.2.1 Poly (ADP-ribose) polymerase inhibitors (PARP inhibitors)

PARP inhibitors trap DNA to destabilize the replication fork and induce cell death through mitotic catastrophe caused by replication stress, which are used to treat BRCA-mutated ovarian cancer, breast cancer, pancreatic cancer. Olaparib’s prototype is excreted in urine by 15%, and its metabolites are excreted in urine. Olaparib was not detectable in the dialysate samples. Pharmacokinetic studies have shown that patients with renal impairment exhibit increased systemic exposure and higher peak concentrations of the PARP inhibitor olaparib, which may progressively increase the risk of hematologic adverse effects (81). A daily dose of 400 mg olaparib, given in two divided doses, was well tolerated by a patient with ESRD receiving hemodialysis, resulting in 16 months of progression-free survival (82).

#### 5.2.2 Anti-angiogenics agents

Monoclonal antibodies are eliminated via catabolism by lysosomal degradation to peptides and amino acids. The high molecular weight of monoclonal antibodies makes it impossible for the kidneys to eliminate them, unless there is a presence of pathologic conditions. And it is not dialyzable. However, the blocking of Vascular endothelial growth factor/Vascular endothelial growth factor receptors pathway may induce hypertension and TMA in ESRD patients. The pharmacokinetic parameters of Bevacizumab observed in a patient receiving this dosage were comparable to those reported in individuals with normal renal function who were administered 10 mg/kg every 14 days (83). Bevacizumab has been also used at a dose of 5 mg/kg every 14 days in hemodialysis patients (84, 85). Sorafenib, Axitinib, Sunitinib, Ramucirumab, Pazopanib are metabolized mainly in the liver and their urinary excretion less than 20%. Pharmacokinetic and clinical assessments have shown that these treatments are effective and well-tolerated in hemodialysis patients with cancer (23, 84–88).

Cabozantinib and its metabolites are excreted in the feces (54%), as well as in the urine (27%). Cabozantinib displays a long-term plasma half-life (-120 h) and accumulates five-fold by day 15 following daily dosing based on AUC. Its AUC was increased by 7–30% in subjects with mild/moderate renal impairment (89). Therefore, the frequency of Cabozantinib use should be reduced in hemodialysis patients. Case report observed that a patient tolerated a regorafenib dose of 40 mg daily for 21 days, the treatment was interrupted after one cycle of treatment because of a septic shock and the worsening of cardiomyopathy (90).

As far as nintedanib, less than 1% is excreted via the kidney and adjustment dose in patients with mild to moderate renal injury is not required. However, Lenvatinib has been associated with a range of renal adverse effects, such as hypertension, focal segmental glomerulosclerosis, and TMA (91). Vandetanib has been found to inhibit certain human renal transporters, resulting in reduced creatinine clearance and enhanced cisplatin-induced nephrotoxicity (92). Therefore, Lenvatinib and Vandetanib should be avoid in patients with residual renal function. Collectively, multicenter retrospective study indicated that anti-angiogenics agents seem to be effective and can be used among patients undergoing hemodialysis. PK assessments were not modified in patients undergoing hemodialysis compared with a population not undergoing dialysis (84, 93, 94).

#### 5.2.3 Epidermal growth factor receptor tyrosine kinases inhibitor (EGFR-TKIs)

EGFR-TKIs are poorly removed by hemodialysis, as over 90% of EGFR-TKIs bind to plasma proteins; these agents are primarily metabolized in the liver and predominantly excreted via bile. Available EGFR inhibitors presently include three monoclonal antibodies (cetuximab, panitumumab and necitumumab) and five small molecule tyrosine kinase inhibitors (TKIs – gefitinib, erlotinib, afatinib, dacomitinib and osimertinib). Cetuximab, panitumumab, gefitinib, and erlotinib have been found to be well-tolerated in patients with ESRD or those undergoing hemodialysis, with no requirement for dose adjustments (23, 95). Three patients with lung cancer undergoing hemodialysis achieved a partial response when treated with afatinib, with no serious adverse events observed, and pharmacokinetic data were consistent with those seen in individuals with normal renal function (96). A multicenter retrospective analysis of 18 patients with EGFR-mutant non-small cell lung cancer treated with EGFR tyrosine kinase inhibitors (TKIs)—including gefitinib, erlotinib, and afatinib—across 22 institutions in Japan reported an objective response rate of 44.4% and a median overall survival of 38.6 months. Dose adjustments were required in five patients (27.8%). Treatment discontinuation due to any cause or specifically attributable to treatment-related adverse events (TRAEs) occurred in 50.0% and 33.3% of patients, respectively. The most frequently reported grade 3 or higher adverse events were neutropenia, thrombocytopenia, anemia, and febrile neutropenia, all of which were controllable with appropriate management (43).

#### 5.2.4 Breakpoint cluster region-Abelson leukemia virus inhibitors

Imatinib is the only approved and widely used breakpoint cluster region-Abelson leukemia (BCR-ABL) inhibitor in clinical practice for gastrointestinal stromal tumors. Following mainly hepatic metabolization via CYP3A4 or CYP3A5, Imatinib mesylate and its metabolites are eliminated mostly in feces due to hepatobiliary excretion, with 13% of excretion in the urine. Pharmacokinetic evaluation in a patient with ESRD undergoing hemodialysis and receiving oral imatinib at a daily dose of 400 mg revealed that hemodialysis had no clinically significant effect on systemic imatinib exposure (23, 97, 98).

#### 5.2.5 Mammalian target of rapamycin inhibitors

Everolimus and Temsirolimus are both extensively metabolized by the liver via CYP3A4 and only less excreted in the urine. A PK study showed no significant differences in PK parameters of temsirolimus between patients undergoing hemodialysis and those without dialysis (99). Everolimus and temsirolimus, indeed, are not dialyzed by commonly used membranes. Case reports and a retrospective analysis involving 11 patients with metastatic renal cell carcinoma and end-stage renal disease on hemodialysis indicated that everolimus therapy is feasible, demonstrating favorable efficacy and no unforeseen adverse effects (23, 43, 100). Ten Japanese patients with metastatic renal cell carcinoma undergoing hemodialysis were treated with temsirolimus, achieving favorable clinical outcomes without unexpected severe adverse events and sustaining a relative dose intensity of 89.5% over the entire study duration (101).

#### 5.2.6 Human epidermal growth factor receptor-monoclonal antibodies

These agents are represented either by monoclonal antibodies (trastuzumab, pertuzumab, Trastuzumab Emtansine – T-DM1) or by TKIs (lapatinib and neratinib). As a recombinant humanized monoclonal antibody, the elimination of Trastuzumab involves clearance of immunoglobulin G through the reticuloendothelial system. Trastuzumab emtasine (T-DM1) elimination half-life (approximately 4 days) is shorter than that for a typical IgG1 antibody (i.e. 2–3 weeks); And due to its high molecular weight, clearance of the drug is not expected to be significantly influenced by renal function (102). No increased toxicity and favorable clinical outcomes have been observed with both trastuzumab and T-DM1 in breast cancer patients undergoing hemodialysis (23, 103). Lapatinib is primarily excreted in the feces, less than 2% of administered oral dose being excreted in the urine. Lapatinib demonstrated efficacy in a breast cancer patient receiving hemodialysis for more than three years, with no substantial increase in toxicity (104).

#### 5.2.7 B-Raf proto-oncogene, serine/threonine kinase inhibitors

Renal excretion of dabrafenib is higher than that of vemurafenib (23% urinary excretion versus 1%) and the risk of accumulation in patients with severe CKD or ESRD is higher, therefore, dabrafenib should be used with greater caution in the setting of renal failure. Vemurafenib, due to prolonged QTc interval, and the combination of dabrafenib with trametinib were administered with dose reductions in patients with ESRD (105–107).

#### 5.2.8 Cyclin-dependent kinase 4/6 inhibitor

More recently, Cyclin-dependent kinase 4/6 (CKD 4/6) inhibitor drugs such as (palbociclib, ribociclib, and abemaciclib) have been introduced for hormone receptor-positive metastatic breast cancers, which enhance the efficacy of antiestrogen drugs. All the CKD 4/6 inhibitors are protein-bound molecules metabolized in the liver by cytochrome P450 isoenzymes and less renal excretion. In patients with severe renal impairment, systemic exposure—as measured by AUC_inf_—was 31% higher, and peak plasma concentration (C_max_) was 15% greater, relative to individuals with normal renal function (108). Furthermore, under steady-state conditions, both the 24-hour area under the concentration–time curve (AUC_inf_) and C_max_ were elevated in hemodialysis-dependent patients receiving a reduced palbociclib starting dose of 100 mg/day, compared with values observed in participants with preserved renal function who received 125 mg/day in a Phase I clinical trial (109). Therefore, dose reduction of palbociclib is recommended for patients undergoing maintenance hemodialysis.

#### 5.2.9 Selective estrogen receptor modulator

Tamoxifen and Anastrozole are mainly excreted in feces and only 9%-14% in the urine. The plasma concentrations of the two drugs were similar in hemodialysis patients compared to non-hemodialysis patients. Exemestane, letrozole, and fulvestrant all had only a small portion excreted unaltered in the urine. Thus, dose adjustments are not required for the use of selective estrogen receptor modulators in patients undergoing hemodialysis (23, 110, 111). Megestrol acetate has demonstrated safety and efficacy in the treatment of metastatic breast cancer and has been shown to improve nutritional status and attenuate systemic inflammation in elderly hemodialysis patients with cachexia (112).

### 5.3 Immunotherapies for malignancies

#### 5.3.1 Immune checkpoint inhibitors

Immune checkpoint inhibitors (ICIs) are humanized or fully human immunoglobulin antibodies. Their elimination occurs primarily via intracellular catabolism through lysosomal degradation following pinocytosis, particularly in organs and tissues enriched with endothelial cells, or via receptor-mediated endocytosis (113). The half-lives of these agents range from 6 to 27 days (114). Pharmacokinetic analysis in patients stratified by estimated glomerular filtration rate (eGFR)—including mild (eGFR: 60–89 mL/min/1.73 m²; n=313), moderate (eGFR: 30–59 mL/min/1.73 m²; n=140), and severe (eGFR: 15–29.89 mL/min/1.73 m²; n=3) renal impairment—revealed no clinically significant differences in nivolumab clearance between patients with renal impairment and those with normal renal function. Therefore, dose adjustment is not recommended in patients with renal impairment (115). Pharmacokinetic analyses further indicated the absence of dialytic clearance (114). Case series report a variety of malignancies treated with different ICIs in ESRD, including melanoma, renal cell carcinoma, Colorectal cancer, RCC, renal cell cancer; NSCLC, non-small cell lung cancer (116). A review of 14 articles on the use of immune checkpoint inhibitors (ICIs) in cancer patients undergoing dialysis encompassed 54 reported cases. Among these patients, 46% (25 individuals) achieved at least a partial response, whereas 35% (19 individuals) experienced disease progression. Immune-related adverse events (irAEs) were observed in 27 individuals (50%), with 75% of these cases classified as grade 1 or 2. Treatment involving corticosteroids was required in 18.5% of patients (117).Another analysis based on 33 articles included 98 cases, of which 80 patients had been diagnosed with ESKD prior to receiving ICIs, while an additional 18 were kidney transplant recipients who experienced allograft rejection following ICI administration and subsequently initiated dialysis while continuing treatment with these agents (116). Overall, 49% of the patients (48 out of 98) developed irAEs. Among these individuals, 15 patients (15%) experienced grade 3 or grade 4 adverse events, including encephalitis and myocarditis, as well as pemphigoid rash. These severe immune-related complications are comparable to those observed in the general population and occur infrequently, with reported incidence rates of 1.3% and less than 1%, respectively (118). Thus, the efficacy and safety profiles of ICIs are comparable between dialysis and non-dialysis patients. Administration of ICIs in patients undergoing dialysis does not lead to a higher risk of developing irAEs (119). However, relatively high frequency of hematologic adverse events was observed in patients with ESKD receiving ICI therapy, which may be related with uremic accumulation and dialysis incompatibility (120). Corticosteroids are recommended as the first-line treatment for ESRD patients experiencing severe irAEs (121, 122).

#### 5.3.2 Chimeric antigen receptor T cell therapy

Chimeric antigen receptor T cell (CAR-T) therapy is a form of adoptive immunotherapy that involves the ex vivo genetic modification of a patient’s T cells to express chimeric antigen receptors specifically directed against tumor-associated antigens. This modification enables the engineered T cells to selectively recognize and target malignant cells. After *in vitro* expansion, these modified T cells are reinfused into the patient, where they induce targeted immune activation and mediate the elimination of tumor cells (123). This therapy has shown remarkable efficacy in treating hematological malignancies such as lymphoma, leukemia, and multiple myeloma, as well as some solid tumors (124). During the CAR T therapy, lymphodepletion is important to allow for proper expansion and survival of CAR T-cell products. Fludarabine and cyclophosphamide are the most common lymphodepleting agents. Fludarabine’s major metabolite, 2-F-ara-A, is primarily excreted in the urine. And approximately 50% to 70% of CTX is excreted by kidneys as mentioned above. Therefore, dose adjudgment is vital for the ESRD patients receiving the lymphodepletion.

lymphodepletion regimen for tisa-cel or brexu-cel is suggested using dose-reduce fludarabine by 50% to 12.5–15 mg/m^2^ with cyclophosphamide maintained at full dose (500 mg/m^2^); and with hemodialysis performed on day 5 and day 3, approximately 12 hours following chemotherapy (125). Hunter et al. recently published their experience with CAR T-cell therapy in two ESRD patients with R/R DLBCL. Their lymphodepletion strategy was elected to dose-reduce cyclophosphamide 40% to 300 mg/m^2^ in their axi-cel patient while maintaining the standard cyclophosphamide dose (300 mg/m^2^) in their liso-cel patient. And the hemodialysis was performed 12 hours after each dose that was given. The adverse effects of CAR T-cell therapy should be monitored in ESRD patients, including cytokine release syndrome, immune effector-associated neurotoxicity syndrome and tumor lysis syndrome (126).

Plasmapheresis or hemofiltration helped quickly clear cytokines, speeded up patient recover, and successfully resolved the severe cytokine release syndrome crisis. Notably, fludarabine toxicity may exacerbate immune effector-associated neurotoxicity syndrome, or that dialysis-dependent patients may experience more severe encephalopathy from any cause due to abnormal renal elimination (127).

Fortunately, lymphodepleting preconditioning—typically essential to support the expansion of adoptively transferred CAR-T cells—is not required and is fundamentally unsuitable for *in vivo* CAR-T approaches. However, as a living therapeutic modality, the *in vivo* pharmacokinetic behavior of CAR-T cells is predominantly determined by immune microenvironmental factors and tumor antigen engagement—not by renal clearance mechanisms. To date, dedicated pharmacokinetic studies, large-scale clinical trials, and formal clinical practice guidelines addressing CAR-T cell therapy in patients with end-stage kidney disease requiring dialysis remain notably lacking. As a result, *in vivo* CAR-T cell therapy holds promise as an immunotherapeutic strategy for patients with end-stage renal disease (ESRD), as it circumvents the need for chemotherapy and enables more effective recruitment of diverse immune mechanisms, which are critical for enhancing therapeutic outcomes (128).

Collectively, the majority of treatment recommendations are based on low-quality evidence and lack support from randomized controlled trials. To date, no authoritative, evidence-based, and comprehensive clinical practice guidelines exist to guide anticancer therapy in patients undergoing hemodialysis. Pharmacokinetic data remain insufficient, particularly for novel anticancer agents such as targeted therapies and immune checkpoint inhibitors, and corresponding pharmacokinetic/pharmacodynamic studies are significantly underdeveloped. Moreover, toxicity management presents significant clinical challenges in this population, attributable to their compromised baseline health status, high burden of comorbidities, diminished tolerance to drug-induced toxicities, and difficulties in both recognizing and mitigating adverse events.

Therefore, clinicians should individualize antineoplastic therapy in patients with ESRD based on available pharmacokinetic and pharmacodynamic data, as well as current clinical recommendations.

## 6 Emerging research directions

### 6.1 Predict the risk of chemotherapy-induced kidney injury in tumor patients through genomic analysis and formulate individualized treatment plans

Utilize artificial intelligence and data analysis to recommend the appropriate dose as well as predict the risk of adverse effects in ESRD patients undergoing chemotherapy.

### 6.2 The development of targeted anti-tumor agents with reduced nephrotoxicity and minimal renal clearance

Platinum-based antineoplastic drugs delivered via O-succinyl chitosan (BSCT) self-assembling nanocarriers or tolfenamic acid-derived prodrug nanoparticles (48), as well as albumin-conjugated doxorubicin utilizing self-assembling dyes (76), represents a promising strategy for improving therapeutic efficacy while mitigating adverse effects. 

### 6.3 Development of individualized chemotherapy regimens for hemodialysis patients guided by pharmacokinetic and pharmacodynamics principles

In the era of precision medicine, accumulating evidence suggests that conventional cytotoxic agents may be viable candidates for therapeutic drug monitoring-guided dose optimization and individualized tailoring of non-selective chemotherapy regimens. Furthermore, mass spectrometry-based methods have been established for the measurement of conventional cytotoxic anticancer agents (129).

### 6.4 The potential contribution of chemotherapy-associated bone disorders—including endocrine therapy and estrogen deprivation—to the pathogenesis and progression of renal osteodystrophy in patients with ESRD

Well-designed clinical studies are needed to evaluate the safety and efficacy of targeted agents such as denosumab and cinacalcet in managing hypercalcemia among uremic patients.

## 7 Conclusion

Although guidelines exist for the use of chemotherapy agents in patients with chronic kidney disease and malignancies, many questions remain unanswered, particularly regarding the pharmacokinetics and pharmacodynamics of these drugs in patients with end-stage renal disease undergoing hemodialysis. Leveraging precision medicine, susceptibility gene screening enables the identification of individuals at elevated risk for kidney injury, thereby supporting early interventions to mitigate progression from chronic kidney disease to end-stage renal disease. The optimal dose of antineoplastic agents is determined on an individual basis according to pharmacokinetic and pharmacodynamic principles to avoid adverse drug reactions resulting from excessive exposure in patients with end-stage renal disease undergoing hemodialysis. Notably, artificial intelligence holds promise as a predictive and early-alert tool for antineoplastic drug–related adverse events in patients with end-stage renal disease undergoing hemodialysis. Furthermore, the development of targeted antineoplastic agents has contributed to reduced renal toxicity and improved intratumoral drug accumulation. Future clinical trials evaluating antineoplastic therapies should prospectively enroll patients undergoing hemodialysis to improve survival outcomes and quality of life.
